# Comparison of safety profiles for dapagliflozin based on EMA and FDA safety issues: Challenges and future of post-marketing surveillance in Korea

**DOI:** 10.1371/journal.pone.0314363

**Published:** 2024-11-22

**Authors:** Suvin Park, Hee-Jin Kim, Heehyun Won, Hui-Eon Lee, Haerin Cho, Nam-Kyong Choi

**Affiliations:** 1 Department of Health Convergence, College of Science and Industry Convergence, Ewha Womans University, Seoul, Korea; 2 Graduate School of Industrial Pharmaceutical Science, College of Pharmacy, Ewha Womans University, Seoul, Korea; Peking University First Hospital, CHINA

## Abstract

Europe, the United States (U.S), and Korea each maintain post-marketing surveillance (PMS) systems to monitor rare or unexpected adverse events. Korea’s PMS mainly involves a re-examination system to identify new adverse events not seen in pre-market trials during the early stages of post-marketing drug use, along with the risk management plan (RMP), a comprehensive strategy using methods like signal detection to regularly assess safety and benefit-risk throughout the drug’s lifecycle. This study compares the post-marketing safety issues associated with dapagliflozin as identified by the European Medicines Agency (EMA), the U.S Food and Drug Administration (FDA), and in Korea. To identify these safety issues, we reviewed the safety concerns listed in the European Union RMP (EU-RMP), adverse events noted in the Warnings and Precautions section of the U.S FDA drug label, and use-result surveillance results detailed in the Korean Ministry of Food and Drug Safety drug label. Additionally, we used Korean Adverse Event Reporting System (KAERS) data to detect safety signals. We manually matched and compared safety issues identified by the EMA and FDA with those recognized in Korea. For safety issues unique to Korea, we compared KAERS signals with the results from use-result surveillance. We compared 17 EMA/FDA safety issues with 38 KAERS signals and 231 results from use-result surveillance. While there was a significant concordance (71%) between the safety issues identified by the EMA/FDA and those in Korea, Korean safety issues had limitations in capturing long-term outcomes and laboratory results. Some safety issues that were initially recognized in the EU-RMP and FDA drug labels were no longer found in the latest documents. To enhance PMS in Korea, it is necessary to establish more specific laws and regulations and develop detailed guidelines that utilize a variety of real-world data and research methodologies to continuously assess causality throughout the product lifecycle.

## Introduction

Drug candidates typically undergo a rigorous screening process that can last 10 to 15 years. However, it is not uncommon for new adverse drug reactions to emerge after the drug is approved. Effective post-marketing surveillance (PMS) by national drug regulatory authorities could prevent or mitigate such incidents [1]. Both the European Medicines Agency (EMA) and the United States (U.S) Food and Drug Administration (FDA) conduct routine and additional pharmacovigilance through spontaneous adverse event (AE) reports and post-marketing studies. Post-Authorization Safety Studies (PASS) are either imposed by the EMA or voluntarily initiated by the marketing authorization holder (MAH) to gather additional information on a drug’s safety or to measure the effectiveness of risk management measures [2]. Postmarketing commitments and requirements (PMR/Cs) refer to the research and clinical trials conducted by the MAH after FDA approval to obtain further information on the product’s safety, effectiveness, or optimal use [3, 4]. Re-examination, introduced in 1995, is a major regulatory requirement for drugs marketed in Korea. Its primary purpose is to observe the usage patterns of new drugs and other products during the early stages of use over a certain period in a broad patient population, which might not have been observed in pre-market clinical trials, to identify new AEs, their occurrence situations, and factors affecting efficacy and safety that were not evident during the drug development and approval process, and to re-evaluate them based on usage records. The MAH must conduct PMS for a period of 4 or 6 years from the date of initial product approval, depending on the specific item. When the surveillance period ends, the MAH must undergo a re-examination by the Korean Ministry of Food and Drug Safety (MFDS), which may result in follow-up measures such as changes to the approved indications and precautions for use based on the re-examination results [5]. In addition to re-examinations, the MAH must regularly report the results of safety evaluations or benefit-risk assessments according to the risk management plan (RMP) submitted at the time of the marketing authorization application. During this process, they can use methods such as signal detection analysis on the collected AE information [6].

Sodium-glucose co-transporter 2 (SGLT2) inhibitors are novel drugs with advantages such as reduced risk of hypoglycemia and promotion of weight loss. Among these, dapagliflozin is particularly noted as a potent and selective SGLT2 inhibitor [7]. Although they are generally considered safe now, serious AEs such as diabetic ketoacidosis (DKA), lower limb amputation, and bladder cancer remain significant concerns. Following its approval, 5 PASS related to dapagliflozin were listed in the European post-authorisation study (EU PAS) Register [8], while the FDA outlined 6 PMR/Cs [9]. In 2019, the MAH completed a 6-year re-examination of dapagliflozin, and the results were updated on the Korean drug label.

In this study, we identify safety issues associated with dapagliflozin in Korea by utilizing results from its re-examination and signals from the Korea Adverse Event Reporting System (KAERS) data. Additionally, we compare these safety issues with those listed as safety concerns (SCs) in the European Union Risk Management Plan (EU-RMP) and with the AEs noted in the Warnings and Precautions (WPs) section of the FDA drug label.

## Materials and methods

This study aims to carry out the following steps: First, we explain the method for reviewing the re-examination results that we have defined as Korean safety issues. Next, we present the method for defining EMA/FDA safety issues and reviewing the AEs of dapagliflozin that have been monitored by EMA and FDA, either currently or in the past. Then, we describe the analysis method for deriving KAERS results, which we define as another type of Korean safety issue. Finally, we explain the standardization and manual matching process for comparing AEs between Korea and EMA/FDA, which use different terminology classification systems (S1 Fig).

### Identifying Korean safety issues: Reviewing the re-examination results

We conducted a review of the re-examination results for dapagliflozin in Korea. According to the ‘Standards on Re-examination of New Drugs’ stipulated by the MFDS, the MAH is required to conduct this PMS study. The MAH typically selects use-result surveillance to collect the necessary PMS data for re-examination applications. This investigation is a non-comparative, prospective, non-interventional regulatory PMS study conducted at medical facilities. Usage cases are collected based on the number of study subjects calculated according to objective and valid evidence, such as the safety information of the product, without specifying conditions for the study subjects [5, 10]. For the re-examination of dapagliflozin, the MAH conducted PMS on 3,027 patients over six years, from November 26, 2013, to November 25, 2019. As a follow-up measure to the re-examination results, the MFDS required the PMS results to be added to the product label. These results are detailed in the ‘Precautions for Use’ section of the dapagliflozin label in Korea [11].

### Identifying and defining EMA/FDA safety issues: Reviewing documents from EMA/FDA websites

In the EMA, post-marketing monitoring requirements are managed through the RMP, which is developed at the time of drug approval. This plan includes risk minimization strategies, PASS, and other pharmacovigilance activities for all major SCs associated with the use of the product [12, 13]. The FDA approves hundreds of label changes annually based on various post-marketing data [14]. The WPs section specifies serious or significant AEs and potential SCs, based on evidence of an association between the drug and the AEs [15]. SCs listed in the EU-RMP were defined as ‘EMA safety Issues,’ while AEs listed in the WPs section of the FDA drug label were defined as ‘FDA safety Issues.’

The European public assessment report (EPAR) includes a summary of all SCs extracted from the EU-RMP [16]. We reviewed 10 EPARs related to dapagliflozin monotherapy, which were posted on the EMA website from December 7, 2012, to March 1, 2023. The initial dapagliflozin EPAR, published on December 7, 2012, marks the start date of our study. ‘EMA Safety Issues’ encompass all SCs mentioned at least once in these documents. Fractures, first identified in the December 7, 2012, EPAR, were not mentioned after August 28, 2019, but continued to be classified as ‘EMA Safety Issues.’ To define ‘FDA Safety Issues,’ we reviewed a total of 18 FDA drug labels related to dapagliflozin monotherapy from January 8, 2014, to September 12, 2023. January 8, 2014, marks both the US approval of dapagliflozin and the date the first drug label was posted on the Drugs@FDA website [17]. We included all AEs mentioned at least once in the WPs section of the drug labels as ‘FDA Safety Issues’. We reviewed all publicly available documents.

**Fig 1:**
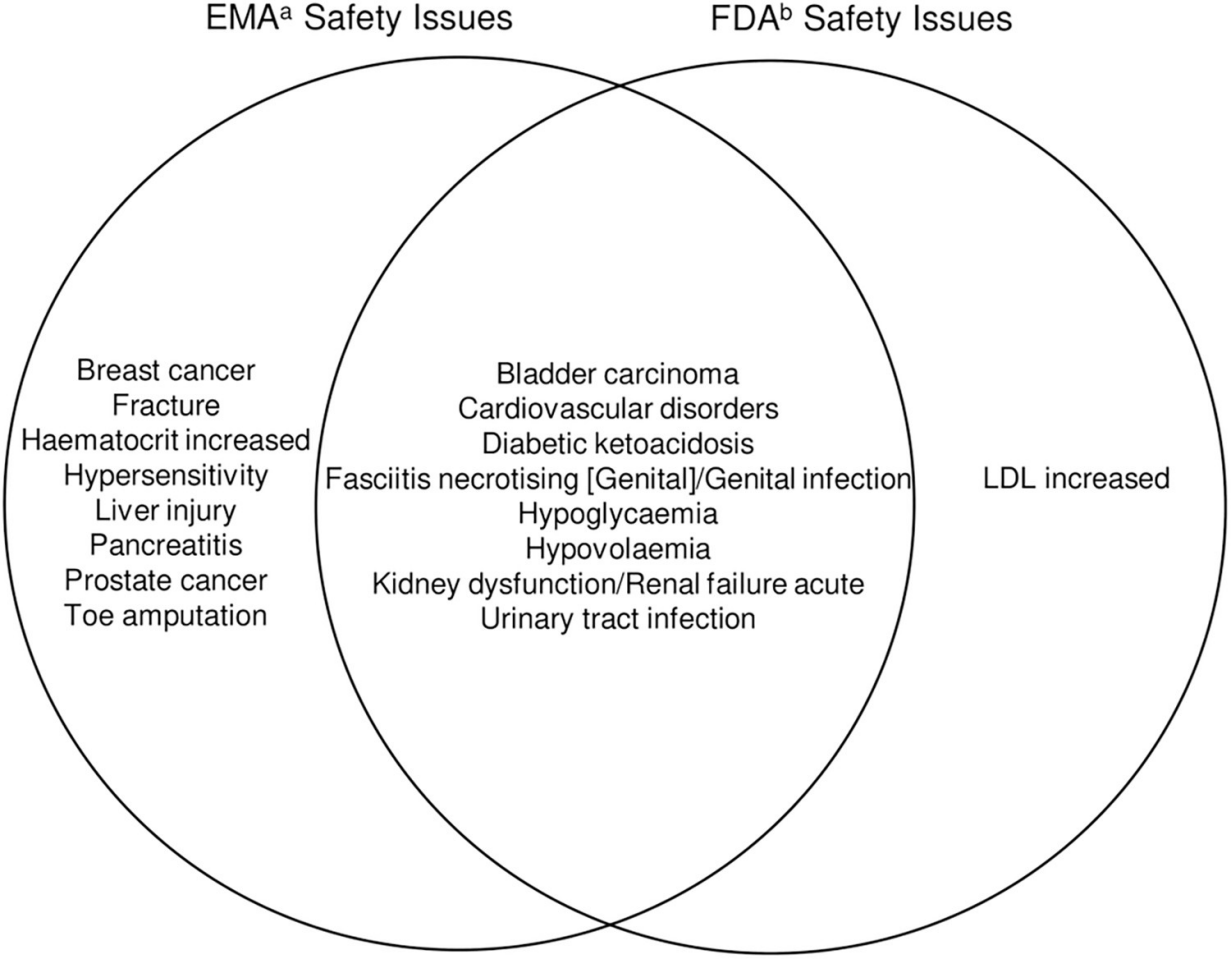
Venn diagram visualizing the EMA and FDA safety issues. EMA, European Medicines Agency; FDA, Food and Drug Administration; LDL, low-density lipoprotein. ^a^Safety concerns in the European Union Risk Management Plan ^b^Adverse events noted in the Warning and Precautions section of the FDA drug label.

Before comparing with the data from Korea, we present Fig 1, which visually compares the AEs identified as risks by the EMA and FDA. This figure aids understanding by simultaneously showing the safety issues recognized as risks by both regulatory bodies and those identified individually by each agency.

### Identifying Korean safety issues: Signal detection using the KAERS data

We used the KAERS data to detect signal information related to AEs reported for dapagliflozin in Korea. Korea first implemented the spontaneous reporting system in 1988, and in 2012, the Korea Institute of Drug Safety and Risk Management (KIDS) was established to perform tasks entrusted by the MFDS. The KIDS collects cases of drug AEs from manufacturers, importers, healthcare professionals, and consumers through KAERS and manages this data in a database. Additionally, the agency utilizes the collected data to detect signals of AEs using statistical methods, and reviews both domestic and international approval information and related literature to provide safety information on pharmaceuticals [18]. The KAERS data can be requested through the MFDS website. It is disclosed only for specific purposes: for drug safety management by universities and research institutions, for public health improvement tasks by national and public institutions, and upon request by a safety management officer for manufacturers and importers. Depending on the scope of the request, data from a minimum of 5 years to a maximum of 10 years can be provided. Considering the approval date of dapagliflozin in Korea on November 26, 2013, we have analyzed data provided by the KIDS from January 1, 2014, to December 31, 2020. This data includes details such as the date of AE reporting, reporter information, and patient details like gender and age, along with the active ingredients of the suspected drugs. All drug names are coded using the Anatomical Therapeutic Chemical Classification System (ATC) codes, and drug AEs are coded according to the Preferred Terms (PTs) from the World Health Organisation Adverse Reactions Terminology (WHO-ART). For the analysis, we created combinations of drugs and AEs using the reported ATC codes and WHO-ART PTs. All technical statistics and data mining analyses were based on these combinations. Data mining can be considered a part of exploratory data analysis and knowledge discovery, aimed at generating hypotheses. Measures of disproportionality use a two-by-two contingency table for each pair of drug events and compare them to the expected number based on reports of the drug and AEs to identify unexpectedly frequent combinations. For example, the proportional reporting ratio (PRR), reporting odds ratio (ROR), and information component (IC) are widely used by the EMA, FDA, and in Korea [19–22]. The drug of interest in this study is dapagliflozin, identified by the ATC code A10BK01. For data mining purposes, the comparison drugs selected included all other oral hypoglycemic agents (OHAs) reported as suspect drugs. An AE was considered a significant signal if it met the following criteria: PRR ≥ 2, ROR ≥ 2, and the lower limit of the 95% confidence interval (CI) of the IC is greater than 0 [23–26]. We also searched for signal information for other SGLT2 inhibitors marketed in Korea, such as ipragliflozin, empagliflozin, and ertugliflozin, to compare the signals detected for dapagliflozin and determine whether these effects are drug-specific or class-specific. Canagliflozin, which was voluntarily withdrawn after being approved in Korea, was excluded from the analysis. Statistical analyses were performed using SAS Enterprise Guide 8.3 (SAS Institute, Cary, NC). Data for this research was accessed initially on 30/12/2022 and most recently on 16/03/2023. The study protocol was approved by the Institutional Review Board of Ewha Womans University (IRB No. ewha-202211-0014-01). Informed consent was waived by the IRB as all data has been completely anonymized.

### Comparison of safety issues: EMA/FDA and Korea

We compared safety issues identified by the EMA and FDA with those recognized in Korea. For Korean-specific safety issues, we compared KAERS signals with use-result surveillance results. Due to differing terminological classification systems for the safety issues, standardization was necessary. To streamline this comparison and encompass a broad spectrum of cases, we converted the safety issues from each dataset to the WHO-ART Preferred Term (PT) level [27]. The initial manual matching of the converted PTs for Korean and EMA/FDA safety issues was conducted by a registered nurse and subsequently reviewed by a health information manager. In cases of disagreement, a final consensus was reached through discussion between the two evaluators.

## Results

### Korean safety issues: Use-result surveillance results

Out of the 3,027 patients aged 18 years or older with type 2 diabetes who were starting treatment with dapagliflozin from November 26, 2013, to November 25, 2019, a total of 805 patients experienced 1,222 AEs, representing 26.6% [11]. S1 File provides a simple translation of the dapagliflozin use-result surveillance results, as listed in the Korean drug label. This file includes a total of 231 AEs, excluding duplicates.

### EMA/FDA safety issues

In total, 17 safety issues were identified by both the EMA and FDA. Among these, 8 were recognized by both agencies. The EMA had 8 unique safety issues, whereas the FDA had 1 unique issue (Fig 1).

### Korean safety issues: KAERS signals

This study analyzed a total of 33,393 reports associated with OHAs from January 1, 2014, to December 31, 2020. Of these, 2,562 reports specifically concerned dapagliflozin, whereas the remaining 30,831 reports involved other OHAs. We identified 38 signals that met all three predefined criteria (Table 1). The signal information results for empagliflozin or ertugliflozin showed that pruritus genital, mouth dry, ketosis, thirst, weight decrease, micturition disorder, micturition frequency, nocturia, polyuria, urinary tract infection, urine abnormal, genital infection, vaginitis, and moniliasis genital overlapped with the signal information results for dapagliflozin. There were no reported cases of ipragliflozin (S2 File).

**Table 1:** Dapagliflozin KAERS signals meeting disproportionality analysis criteria.

AEs		Number of AE reports		Disproportionality analysis			
		Dapagliflozin	All other OHAs	PRR	ROR	IC	*χ* ^2^
Skin and appendages disorders							
	Pruritus genital	166	216	10.62	11.29	2.42	826.60
Musculo-skeletal system disorders							
	Muscle weakness	10	45	3.07	3.08	0.59	11.45
Central & peripheral nervous system disorders							
	Dysaesthesia	4	2	27.65	27.69	1.71	34.25
	Hypoaesthesia	25	35	9.87	9.96	1.98	116.49
Vision disorders							
	Retinal disorder	8	39	2.46	2.47	0.23	5.80
Hearing and vestibular disorders							
	Tinnitus	6	25	3.32	3.32	0.49	7.84
Psychiatric disorders							
	Appetite increased	14	55	3.52	3.53	0.84	20.16
Gastro-intestinal system disorders							
	Increased stool frequency	3	3	13.82	13.84	1.27	17.85
	Mouth dry	65	244	3.68	3.75	1.26	101.12
Liver and biliary system disorders							
	Gallbladder disorder	4	5	11.06	11.07	1.31	20.34
	Liver fatty	9	14	8.89	8.91	1.54	38.37
	SGOT increased	10	61	2.27	2.27	0.24	6.09
Metabolic and nutritional disorders							
	Dehydration	9	16	7.78	7.80	1.44	34.03
	Ketosis	14	24	8.06	8.10	1.63	54.77
	Polydipsia	12	2	82.94	83.33	2.59	138.83
	Thirst	70	102	9.49	9.73	2.19	316.60
	Weight decrease	214	387	7.64	8.25	2.17	808.57
Cardiovascular disorders, general							
	Hypotension postural	7	24	4.03	4.04	0.75	12.37
Vascular (extracardiac) disorders							
	Transient ischaemic attack	4	8	6.91	6.92	0.99	13.49
Urinary system disorders							
	Cystitis	35	74	6.54	6.61	1.72	111.79
	Dysuria	24	64	5.18	5.22	1.40	59.08
	Micturition disorder	6	20	4.15	4.15	0.72	11.03
	Micturition frequency	113	169	9.24	9.62	2.25	501.54
	Nocturia	44	31	19.62	19.95	2.59	322.02
	Polyuria	106	82	17.87	18.60	2.72	739.96
	Pyelonephritis	10	23	6.01	6.03	1.26	29.14
	Urethritis	6	10	8.29	8.31	1.31	24.07
	Urinary incontinence	8	32	3.46	3.46	0.64	11.18
	Urinary retention	10	9	15.36	15.42	1.94	63.62
	Urinary tract infection	39	68	7.93	8.04	1.92	150.48
	Urine abnormal	15	49	4.23	4.25	1.06	28.39
Reproductive disorders, male							
	Genital infection	36	10	49.76	50.46	2.91	374.42
Reproductive disorders, female							
	Leukorrhoea	8	1	110.59	110.93	2.41	96.55
	Menstrual disorder	3	6	6.91	6.92	0.86	10.12
	Vaginitis	65	99	9.08	9.29	2.14	283.20
Body as a whole—general disorders							
	Asthenia	41	271	2.09	2.11	0.51	20.45
	Leg pain	11	59	2.24	2.24	0.23	6.40
Resistance mechanism disorders							
	Moniliasis genital	9	9	13.82	13.87	1.83	53.56

AE, adverse event; IC, information component; KAERS, Korea adverse event reporting system; OHAs, oral hypoglycaemic agents; PRR, proportional reporting ratio; ROR, reporting odds ratio; SGOT, serum glutamic oxaloacetic transaminase.

### Comparison of safety issues: EMA/FDA and Korea

**Fig 2:**
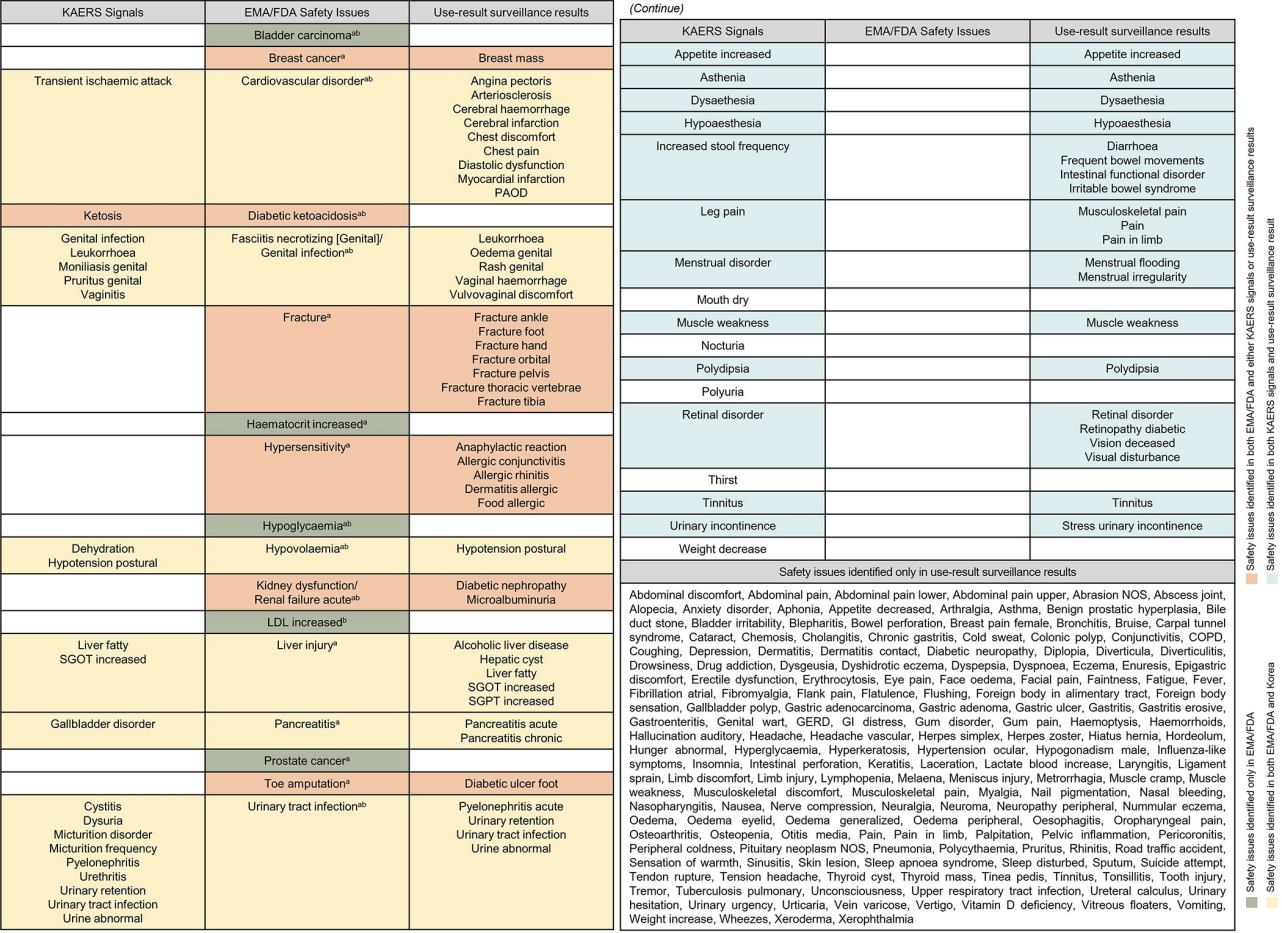
Matching the 17 EMA/FDA safety issues with Korean safety issues. AE, adverse event; COPD, Chronic obstructive pulmonary disease; EMA, European Medicines Agency; FDA, Food and Drug Administration; GERD, gastroesophageal reflux disease; GI, gastrointestinal; KAERS, Korea adverse event reporting system; LDL, low-density lipoprotein; NOS, not otherwise specified; PAOD, Peripheral arteriosclerotic occlusive disease; SGOT, serum glutamic oxaloacetic; SGPT, serum glutamic pyruvate transaminase. ^a^Safety concerns in the European Union Risk Management Plan. ^b^Adverse events noted in the Warning and Precautions section of the FDA drug label.

Among the 17 safety issues identified by the EMA/FDA, six—cardiovascular disorder, fasciitis necrotizing (genital)/genital infection, hypovolaemia, liver injury, pancreatitis, and urinary tract infection—were confirmed in both KAERS signals and use-result surveillance results, indicated in yellow. Of the remaining 11 EMA/FDA issues, DKA was identified solely in KAERS signals, while breast cancer, fracture, hypersensitivity, kidney dysfunction/renal failure acute, and toe amputation were exclusively documented in the use-result surveillance results, highlighted in peach. Additionally, five issues—bladder carcinoma, haematocrit increased, hypoglycaemia, low-density lipoprotein (LDL) increased, and prostate cancer—were not identified in either dataset, marked in green. There are 17 KAERS signals and 189 use-result surveillance results that do not correspond with any EMA/FDA safety issues. Among these, 12 KAERS signals coincided with findings from the use-result surveillance, noted in sky blue. Furthermore, 168 safety issues identified exclusively in the use-result surveillance results did not correspond with any EMA/FDA safety issues or KAERS signals, as shown in Fig 2.

## Discussion

We conducted a comparison of the post-marketing safety issues associated with a specific drug, as identified by the EMA, FDA and in Korea. To identify these safety issues, we reviewed the SCs listed in the EU-RMP, AEs noted in the WPs section of the FDA drug label, and use-result surveillance results detailed in the MFDS drug label. Additionally, we utilized KAERS data to detect relevant signals. Out of the 17 EMA/FDA-identified safety issues, 12 also appeared in Korean data, either through KAERS signals or use-result surveillance results. Notably, six issues were confirmed by both KAERS signals and use-result surveillance results, emphasizing their significance. Conversely, only one issue appeared solely in KAERS signals, while five were exclusively noted in use-result surveillance results.

We particularly focused on ketosis detected from the KAERS signal, an important identified risk where post-approval case reports have triggered safety warnings from both the EMA and FDA regarding DKA [10, 27]. DKA is currently still considered a risk in the most recent EPAR and FDA label documents. A recent study found that real-world incidence of DKA in type 1 diabetes patients using SGLT2 inhibitors off-label is higher than what was expected from clinical trials [28]. This underscores the importance of these findings in the context of KAERS. Furthermore, we matched the breast mass identified in the use-result surveillance results with the breast cancer, an EMA safety issue. Both the EMA and FDA noted a breast cancer imbalance observed in premarketing trials [29]. Consequently, EMA has imposed a PASS (category 3) as part of the pharmacovigilance plan to assess the important potential risk of breast cancer, with this study expected to conclude in December 2024 [30]. On the other hand, bladder carcinoma, haematocrit increased, hypoglycaemia, LDL increased, and prostate cancer were not identified in the Korean safety issues. The data from Korea, based on anecdotal reports, pose challenges in capturing long-term outcomes. This limitation underscores the need for extensive research and analysis to evaluate the association between specific AEs and a drug.

Several issues previously recognized by the EMA or FDA, including fracture, haematocrit increased, hypersensitivity, hypoglycaemia (the latter currently removed only by the EMA), kidney dysfunction/renal failure acute, LDL increased, liver injury, and pancreatitis, are no longer listed in the current version of the EU-RMP or the WPs section. Fractures, hypersensitivity, and kidney dysfunction/renal failure acute were identified through use-result surveillance, while liver injury and pancreatitis were identified through both use-result surveillance and KAERS. The following issues, excluding pancreatitis and hypersensitivity, were initially included in the documentation as risks based on clinical trial results. Pancreatitis was first identified as a risk through pharmacovigilance activities and clinical reports, while hypersensitivity was noted through spontaneous post-marketing reports. The eight safety issues considered important potential risks were removed from the documentation based on updated results from the Dapagliflozin Effect on Cardiovascular Events–Thrombolysis in Myocardial Infarction 58 (DECLARE-TIMI 58) study [31–41].

We reviewed the documents to identify cases where specific risks were added to and subsequently modified. There were differences in the update times of documents related to kidney dysfunction/renal failure acute. Kidney dysfunction/renal failure acute was identified as an important potential risk in pre-marketing clinical trials and included in the initial documentation. The EMA later removed this risk based on the Dapagliflozin and Prevention of Adverse Outcomes in Chronic Kidney Disease (DAPA-CKD) and DECLARE-TIMI 58 studies, updating the EPAR on September 2, 2021. In contrast, the FDA reviewed data from the Dapagliflozin and Prevention of Adverse Outcomes in Heart Failure (DAPA-HF) and DECLARE-TIMI 58 studies, resulting in an update on May 5, 2020 [32, 34, 42–45]. These differences suggest that, while there was high concordance in the final decisions of both agencies regarding the same safety issues, the timing and manner of regulatory actions varied. The decisions can significantly differ based on each regulatory authority’s decision-making processes, data interpretation, and perception of the risk’s significance.

The use of real-world evidence (RWE) in supporting regulatory decision-making for pharmaceuticals is gaining significant global attention [46]. Consequently, regulatory agencies worldwide are issuing various guidance documents on RWE, and the MFDS is also improving several systems on post-marketing in response. The MFDS is in the process of phasing out the re-examination system, which is rarely used globally, by 2032 and integrating it with the RMP system that has been implemented since 2015. The RMP includes a comprehensive safety management strategy covering the entire lifecycle of pharmaceuticals [25]. In 2020, the MFDS amended regulations to allow the use of big data from healthcare information in post-market drug safety surveillance [10, 47]. Additionally, in 2021, the MFDS released guidelines outlining methods for utilizing various healthcare data, such as health insurance claims and medical records from hospitals and clinics, in drug safety evaluations, providing specific research methodologies [48]. However, there are several practical challenges in implementing studies, including restricted access to national claims data and limitations on using data collected from healthcare facilities. To enhance the activation of PMS research utilizing real-world data (RWD), it is essential to establish more specific laws and regulations, develop detailed guidelines, and foster better cooperation among all stakeholders involved.

Our study has several limitations. First, the process of matching safety issues was conducted manually, which could introduce subjectivity due to the absence of clear classification criteria. However, efforts to minimize misclassification were made through thorough consultations between two experts and an extensive literature review. Secondly, MAHs are required to report AEs collected during the PMS period to KAERS, which creates potential overlaps between our use-result surveillance results and the data reported to KAERS. Lastly, the signals detected from KAERS and the use-result surveillance results do not imply a causal relationship between dapagliflozin and AEs. Consequently, further pharmacoepidemiological studies using population-based databases are essential to explore the potential associations between new safety information and dapagliflozin.

## Conclusions

Alignment between the EMA/FDA and Korean safety issues was substantial (71%). However, Korean data has limitations in capturing long-term outcomes and laboratory results. Some of these issues were initially recognized in the EU-RMP and FDA drug labels but have been removed in the current versions of these documents. To enhance PMS in Korea, it is necessary to establish more specific laws and regulations and develop detailed guidelines that utilize a variety of RWD and research methodologies to continuously assess causality throughout the product lifecycle.

## Supporting information

S1 Fig Research methodology overview. AE, adverse event; EMA, European Medicines Agency; EPAR, European public assessment report; RMP, Risk Management Plan; FDA, Food and Drug Administration; IC, information component; KAERS, Korea adverse event reporting system; KIDS, Korea Institute of Drug Safety and Risk Management; MAH, marketing authorization holder; MFDS, Ministry of Food and Drug Safety; OHA, oral hypoglycaemic agent; PRR, proportional reporting ratio; PT, preferred term; ROR, reporting odds ratio; WHO-ART, World Health Organization Adverse Reactions Terminology.
(TIF)

S1 File Dapagliflozin use-result surveillance results from the Korean drug label. AE, adverse event; ADR, adverse drug reaction; COPD, Chronic obstructive pulmonary disease; GERD, gastroesophageal reflux disease; GI, gastrointestinal; NOS, not otherwise specified; PAOD, Peripheral arteriosclerotic occlusive disease; SGOT, serum glutamic oxaloacetic; SGPT, serum glutamic pyruvate transaminase; WHO-ART, World Health Organization adverse reaction terminology.
(DOCX)

S2 File Empagliflozin and ertugliflozin KAERS signals meeting disproportionality analysis criteria. AE, adverse event; IC, information component; KAERS, Korea adverse event reporting system; NOS, not otherwise specified; OHAs, oral hypoglycaemic agents; PRR, proportional reporting ratio; ROR, reporting odds ratio.
(DOCX)

## Abbreviations

KAERS: Korea Adverse Event Reporting System
MAH: marketing authorization holder
MFDS: Ministry of Food and Drug Safety
PMS: post-marketing surveillance
RMP: risk management plan
SC: safety concern; WP, Warnings and Precaution
