# Supplementary figures and images for: Comparison of safety profiles for dapagliflozin based on EMA and FDA safety issues: Challenges and future of post-marketing surveillance in Korea
Source: PLoS One. 2024 Nov 22;19(11):e0314363. doi: 10.1371/journal.pone.0314363 (PMC11584137; doi:10.1371/journal.pone.0314363)

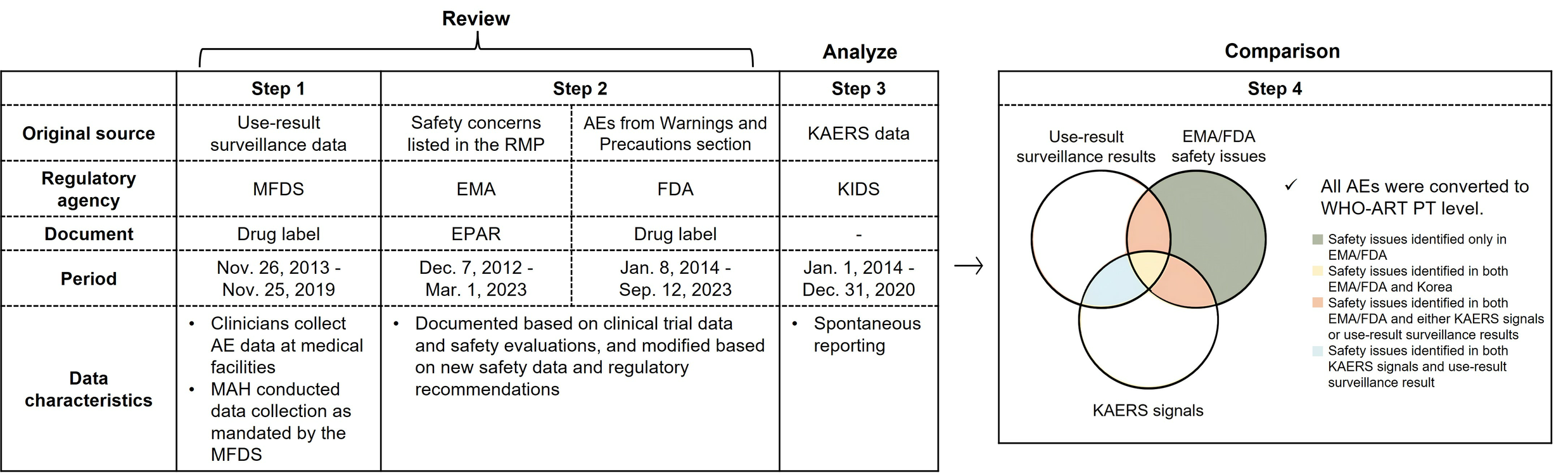

Supplement: S1 Fig — AE, adverse event; EMA, European Medicines Agency; EPAR, European public assessment report; RMP, Risk Management Plan; FDA, Food and Drug Administration; IC, information component; KAERS, Korea adverse event reporting system; KIDS, Korea Institute of Drug Safety and Risk Management; MAH, marketing authorization holder; MFDS, Ministry of Food and Drug Safety; OHA, oral hypoglycaemic agent; PRR, proportional reporting ratio; PT, preferred term; ROR, reporting odds ratio; WHO-ART, World Health Organization Adverse Reactions Terminology. (TIF) [file pone.0314363.s001.tif]
