# Supplementary material for: Comparison of safety profiles for dapagliflozin based on EMA and FDA safety issues: Challenges and future of post-marketing surveillance in Korea
Source: PLoS One. 2024 Nov 22;19(11):e0314363. doi: 10.1371/journal.pone.0314363 (PMC11584137; doi:10.1371/journal.pone.0314363)
Supplement: S1 File — AE, adverse event; ADR, adverse drug reaction; COPD, Chronic obstructive pulmonary disease; GERD, gastroesophageal reflux disease; GI, gastrointestinal; NOS, not otherwise specified; PAOD, Peripheral arteriosclerotic occlusive disease; SGOT, serum glutamic oxaloacetic; SGPT, serum glutamic pyruvate transaminase; WHO-ART, World Health Organization adverse reaction terminology. (DOCX) [file pone.0314363.s002.docx]

**Supporting Information 1**

S1 file: Dapagliflozin use-result surveillance results from the Korean drug label

| The frequency of occurrence | WHO-ART SOC | Serious AEs unrelated to causality | Serious ADRs where causality cannot be excluded | Unexpected AEs unrelated to causality | Unexpected ADRs where causality cannot be excluded |
| --- | --- | --- | --- | --- | --- |
|  |  | 1.59% (48/3,027 person, 52 cases) | 0.17% (5/3,027 person, 5 cases) | 15.73% (476/3,027 person, 624 cases) | 3.67% (111/3,027 person, 129 cases) |
| Rare (less than 0.01-0.1%) | Skin and appendages disorders | - | - | Alopecia, Dermatitis, Dermatitis allergic, Dermatitis contact, Dyshidrotic eczema, Eczema, Genital wart, Hyperkeratosis, Nail pigmentation, Nummular eczema, Rash genital, Skin lesion, Tinea pedis, Xeroderma | Cold sweat, Dermatitis, Hyperkeratosis, Skin lesion, Urticaria, Xeroderma |
|  | Musculo-skeletal system disorders | Fracture ankle, Fracture hand, Fracture pelvis, Fracture thoracic vertebrae, Fracture tibia, Ligament sprain, Meniscus injury | - | Abrasion NOS, Fibromyalgia, Fracture ankle, Fracture foot, Fracture hand, Fracture orbital, Fracture pelvis, Fracture thoracic vertebrae, Fracture tibia, Laceration, Limb discomfort, Limb injury, Meniscus injury, Muscle cramp, Musculoskeletal discomfort, Osteopenia, Tendon rupture | Muscle weakness |
|  | Central & peripheral nervous system disorders | Nerve compression, Dysaesthesia | - | Aphonia, Diabetic neuropathy, Facial pain, Faintness, Headache vascular, Nerve compression, Neuralgia, Neuropathy peripheral, Tension headache, Tremor, Unconsciousness, Vertigo |  |
|  | Vision disorders | Keratitis | - | Allergic conjunctivitis, Blepharitis, Cataract, Chemosis, Conjunctivitis, Diplopia, Eye pain, Hordeolum, Hypertension ocular, Keratitis, Retinal disorder, Vision decreased, Visual disturbance, Vitreous floaters |  |
|  | Special senses other, disorders | - | - | Dysgeusia | Dysgeusia |
|  | Psychiatric disorders | Depression, Drug addiction, Suicide attempt | - | Anxiety disorder, Depression, Drug addiction, Erectile dysfunction, Hallucination auditory, drowsiness, Sleep disturbed, Suicide attempt | Anxiety disorder, Appetite increased |
|  | Gastro-intestinal system disorders | Abdominal pain upper, Bowel perforation, Diverticula, Diverticulitis, GERD, Pancreatitis acute, Pancreatitis chronic | - | Chronic gastritis, Colonic polyp, Diverticula, Diverticulitis, Epigastric discomfort, Flatulence, Foreign body in alimentary tract, Frequent bowel movements, Gastritis erosive, Gastroenteritis, Gum disorder, Gum pain, Haemorrhoids, Hiatus hernia, Intestinal functional disorder, Intestinal perforation, Irritable bowel syndrome, Melaena, Pancreatitis acute, Pancreatitis chronic, Pericoronitis, Tooth injury | Abdominal discomfort, Abdominal pain, Abdominal pain lower, Frequent bowel movements, Gastritis, GI distress, Oesophagitis |
|  | Liver and biliary system disorders | Alcoholic liver disease, Bile duct stone, Cholangitis | - | Alcoholic liver disease, Bile duct stone, Cholangitis |  |
|  | Metabolic and nutritional disorders | Hyperglycaemia | Hyperglycaemia | Diabetic ulcer foot, Lactate blood increase, Thyroid mass, Vitamin D deficiency, Xerophthalmia | Hyperglycaemia, Lactate blood increase, Weight increase |
|  | Endocrine disorders | - | - | Hypogonadism male, Pituitary neoplasm NOS | Pituitary neoplasm NOS |
|  | Cardiovascular disorders, general | - | - | Diastolic dysfunction | Hypotension postural |
|  | Myo-, endo-, pericardial & valve disorders | Angina pectoris, Myocardial infarction | - | Angina pectoris, Myocardial infarction |  |
|  | Heart rate and rhythm disorders | - | - | Fibrillation atrial |  |
|  | Vascular (extracardiac) disorders | Cerebral haemorrhage, Cerebral infarction, PAOD | - | Arteriosclerosis, Cerebral haemorrhage, Cerebral infarction, Flushing, PAOD, Peripheral coldness, Vein varicose |  |
|  | Respiratory system disorders | COPD, Haemoptysis, Pneumonia, Sinusitis | - | Allergic rhinitis, Asthma, Bronchitis, COPD, Haemoptysis, Laryngitis, Nasal bleeding, Oropharyngeal pain, Pneumonia, Rhinitis, Sinusitis, Sleep apnoea syndrome, Sputum, Tonsillitis, Wheezes |  |
|  | White cell and reticulo-endothelial system disorders | - | - | Lymphopenia |  |
|  | Platelet, bleeding & clotting disorders | - | - | Bruise |  |
|  | Urinary system disorders | Urinary tract infection | Pyelonephritis acute, Urinary tract infection | Bladder irritability, Diabetic nephropathy, Enuresis, Face oedema, Microalbuminuria, Stress urinary incontinence, Ureteral calculus, Urinary hesitation, Urinary urgency | Enuresis, Urine abnormal, Urinary urgency |
|  | Reproductive disorders, male | - | - | Benign prostatic hyperplasia |  |
|  | Reproductive disorders, female | Pelvic inflammation | - | Breast mass, Breast pain female, Menstrual flooding, Metrorrhagia, Pelvic inflammation, Vaginal haemorrhage, Vulvovaginal discomfort | Menstrual irregularity, Vulvovaginal discomfort |
|  | Neoplasms | Gastric adenocarcinoma | - | Erythrocytosis, Gastric adenocarcinoma, Gastric adenoma, Hepatic cyst, Neuroma, Polycythaemia, Thyroid cyst |  |
|  | Body as a whole - general disorders | Anaphylactic reaction, Asthenia, Pain, Tuberculosis pulmonary | - | Anaphylactic reaction, Carpal tunnel syndrome, Fever, Flank pain, Foreign body sensation, Influenza-like symptoms, Oedema, Oedema eyelid, Oedema genital, Sensation of warmth, Tuberculosis pulmonary | Chest pain, Fatigue, Foreign body sensation, Oedema, Oedema genital, Pain |
|  | Resistance mechanism disorders | - | - | Abscess joint, Herpes simplex, Herpes zoster, Otitis media | Herpes simplex |
|  | Secondary terms - events | Road traffic accident | - | Non-drug allergy, Road traffic accident | Non-drug allergy |
| Infrequent (less than 0.1-1%) | Skin and appendages disorders | - | - | Cold sweat, Pruritus, Urticaria | Pruritus |
|  | Musculo-skeletal system disorders | - | - | Arthralgia, Ligament sprain, Muscle weakness, Musculoskeletal pain, Myalgia, Osteoarthritis |  |
|  | Central & peripheral nervous system disorders | - | - | Dysaesthesia, Headache, Hypoaesthesia | Headache |
|  | Vision disorders | - | - | Retinopathy diabetic |  |
|  | Hearing and vestibular disorders | - | - | Tinnitus |  |
|  | Psychiatric disorders | - | - | Appetite decreased, Appetite increased, Hunger abnormal, Insomnia | Appetite decreased, Hunger abnormal |
|  | Gastro-intestinal system disorders | - | - | Abdominal discomfort, Abdominal pain, Abdominal pain lower, Abdominal pain upper, Diarrhoea, Dyspepsia, Enteritis, Gastric ulcer, Gastritis, GERD, GI distress, Nausea, Oesophagitis, Vomiting | Diarrhoea, Dyspepsia, Vomiting, Nausea |
|  | Liver and biliary system disorders | - | - | SGPT increased, SGOT increased, Gallbladder polyp, Liver fatty |  |
|  | Metabolic and nutritional disorders | - | - | Hyperglycaemia, Polydipsia, Weight increase | Polydipsia |
|  | Cardiovascular disorders, general | - | - | Hypotension postural |  |
|  | Heart rate and rhythm disorders | - | - | Palpitation | Palpitation |
|  | Respiratory system disorders | - | - | Coughing, Dyspnoea, Nasopharyngitis, Upper respiratory tract infection |  |
|  | Urinary system disorders | Pyelonephritis acute | - | Urine abnormal, Urinary retention | Urinary retention |
|  | Reproductive disorders, female | - | - | Leukorrhoea, Menstrual irregularity | Leukorrhoea |
|  | Body as a whole - general disorders | Chest pain | - | Asthenia, Chest discomfort, Chest pain, Fatigue, Oedema generalized, Oedema peripheral, Pain, Pain in limb | Asthenia |
| The frequency of occurrence | WHO-ART SOC | Serious AEs unrelated to causality | Serious ADRs where causality cannot be excluded | Unexpected AEs unrelated to causality | Unexpected ADRs where causality cannot be excluded |
|  |  | 1.59% (48/3,027 person, 52 cases) | 0.17% (5/3,027 person, 5 cases) | 15.73% (476/3,027 person, 624 cases) | 3.67% (111/3,027 person, 129 cases) |
| Rare (less than 0.01-0.1%) | Skin and appendages disorders | - | - | Alopecia, Dermatitis, Dermatitis allergic, Dermatitis contact, Dyshidrotic eczema, Eczema, Genital wart, Hyperkeratosis, Nail pigmentation, Nummular eczema, Rash genital, Skin lesion, Tinea pedis, Xeroderma | Cold sweat, Dermatitis, Hyperkeratosis, Skin lesion, Urticaria, Xeroderma |
|  | Musculo-skeletal system disorders | Fracture ankle, Fracture hand, Fracture pelvis, Fracture thoracic vertebrae, Fracture tibia, Ligament sprain, Meniscus injury | - | Abrasion NOS, Fibromyalgia, Fracture ankle, Fracture foot, Fracture hand, Fracture orbital, Fracture pelvis, Fracture thoracic vertebrae, Fracture tibia, Laceration, Limb discomfort, Limb injury, Meniscus injury, Muscle cramp, Musculoskeletal discomfort, Osteopenia, Tendon rupture | Muscle weakness |
|  | Central & peripheral nervous system disorders | Nerve compression, Dysaesthesia | - | Aphonia, Diabetic neuropathy, Facial pain, Faintness, Headache vascular, Nerve compression, Neuralgia, Neuropathy peripheral, Tension headache, Tremor, Unconsciousness, Vertigo |  |
|  | Vision disorders | Keratitis | - | Allergic conjunctivitis, Blepharitis, Cataract, Chemosis, Conjunctivitis, Diplopia, Eye pain, Hordeolum, Hypertension ocular, Keratitis, Retinal disorder, Vision decreased, Visual disturbance, Vitreous floaters |  |
|  | Special senses other, disorders | - | - | Dysgeusia | Dysgeusia |
|  | Psychiatric disorders | Depression, Drug addiction, Suicide attempt | - | Anxiety disorder, Depression, Drug addiction, Erectile dysfunction, Hallucination auditory, drowsiness, Sleep disturbed, Suicide attempt | Anxiety disorder, Appetite increased |
|  | Gastro-intestinal system disorders | Abdominal pain upper, Bowel perforation, Diverticula, Diverticulitis, GERD, Pancreatitis acute, Pancreatitis chronic | - | Chronic gastritis, Colonic polyp, Diverticula, Diverticulitis, Epigastric discomfort, Flatulence, Foreign body in alimentary tract, Frequent bowel movements, Gastritis erosive, Gastroenteritis, Gum disorder, Gum pain, Haemorrhoids, Hiatus hernia, Intestinal functional disorder, Intestinal perforation, Irritable bowel syndrome, Melaena, Pancreatitis acute, Pancreatitis chronic, Pericoronitis, Tooth injury | Abdominal discomfort, Abdominal pain, Abdominal pain lower, Frequent bowel movements, Gastritis, GI distress, Oesophagitis |
|  | Liver and biliary system disorders | Alcoholic liver disease, Bile duct stone, Cholangitis | - | Alcoholic liver disease, Bile duct stone, Cholangitis |  |
|  | Metabolic and nutritional disorders | Hyperglycaemia | Hyperglycaemia | Diabetic ulcer foot, Lactate blood increase, Thyroid mass, Vitamin D deficiency, Xerophthalmia | Hyperglycaemia, Lactate blood increase, Weight increase |
|  | Endocrine disorders | - | - | Hypogonadism male, Pituitary neoplasm NOS | Pituitary neoplasm NOS |
|  | Cardiovascular disorders, general | - | - | Diastolic dysfunction | Hypotension postural |
|  | Myo-, endo-, pericardial & valve disorders | Angina pectoris, Myocardial infarction | - | Angina pectoris, Myocardial infarction |  |
|  | Heart rate and rhythm disorders | - | - | Fibrillation atrial |  |
|  | Vascular (extracardiac) disorders | Cerebral haemorrhage, Cerebral infarction, PAOD | - | Arteriosclerosis, Cerebral haemorrhage, Cerebral infarction, Flushing, PAOD, Peripheral coldness, Vein varicose |  |
|  | Respiratory system disorders | COPD, Haemoptysis, Pneumonia, Sinusitis | - | Allergic rhinitis, Asthma, Bronchitis, COPD, Haemoptysis, Laryngitis, Nasal bleeding, Oropharyngeal pain, Pneumonia, Rhinitis, Sinusitis, Sleep apnoea syndrome, Sputum, Tonsillitis, Wheezes |  |
|  | White cell and reticulo-endothelial system disorders | - | - | Lymphopenia |  |
|  | Platelet, bleeding & clotting disorders | - | - | Bruise |  |
|  | Urinary system disorders | Urinary tract infection | Pyelonephritis acute, Urinary tract infection | Bladder irritability, Diabetic nephropathy, Enuresis, Face oedema, Microalbuminuria, Stress urinary incontinence, Ureteral calculus, Urinary hesitation, Urinary urgency | Enuresis, Urine abnormal, Urinary urgency |
|  | Reproductive disorders, male | - | - | Benign prostatic hyperplasia |  |
|  | Reproductive disorders, female | Pelvic inflammation | - | Breast mass, Breast pain female, Menstrual flooding, Metrorrhagia, Pelvic inflammation, Vaginal haemorrhage, Vulvovaginal discomfort | Menstrual irregularity, Vulvovaginal discomfort |
|  | Neoplasms | Gastric adenocarcinoma | - | Erythrocytosis, Gastric adenocarcinoma, Gastric adenoma, Hepatic cyst, Neuroma, Polycythaemia, Thyroid cyst |  |
|  | Body as a whole - general disorders | Anaphylactic reaction, Asthenia, Pain, Tuberculosis pulmonary | - | Anaphylactic reaction, Carpal tunnel syndrome, Fever, Flank pain, Foreign body sensation, Influenza-like symptoms, Oedema, Oedema eyelid, Oedema genital, Sensation of warmth, Tuberculosis pulmonary | Chest pain, Fatigue, Foreign body sensation, Oedema, Oedema genital, Pain |
|  | Resistance mechanism disorders | - | - | Abscess joint, Herpes simplex, Herpes zoster, Otitis media | Herpes simplex |
|  | Secondary terms - events | Road traffic accident | - | Non-drug allergy, Road traffic accident | Non-drug allergy |
| Infrequent (less than 0.1-1%) | Skin and appendages disorders | - | - | Cold sweat, Pruritus, Urticaria | Pruritus |
|  | Musculo-skeletal system disorders | - | - | Arthralgia, Ligament sprain, Muscle weakness, Musculoskeletal pain, Myalgia, Osteoarthritis |  |
|  | Central & peripheral nervous system disorders | - | - | Dysaesthesia, Headache, Hypoaesthesia | Headache |
|  | Vision disorders | - | - | Retinopathy diabetic |  |
|  | Hearing and vestibular disorders | - | - | Tinnitus |  |
|  | Psychiatric disorders | - | - | Appetite decreased, Appetite increased, Hunger abnormal, Insomnia | Appetite decreased, Hunger abnormal |
|  | Gastro-intestinal system disorders | - | - | Abdominal discomfort, Abdominal pain, Abdominal pain lower, Abdominal pain upper, Diarrhoea, Dyspepsia, Enteritis, Gastric ulcer, Gastritis, GERD, GI distress, Nausea, Oesophagitis, Vomiting | Diarrhoea, Dyspepsia, Vomiting, Nausea |
|  | Liver and biliary system disorders | - | - | SGPT increased, SGOT increased, Gallbladder polyp, Liver fatty |  |
|  | Metabolic and nutritional disorders | - | - | Hyperglycaemia, Polydipsia, Weight increase | Polydipsia |
|  | Cardiovascular disorders, general | - | - | Hypotension postural |  |
|  | Heart rate and rhythm disorders | - | - | Palpitation | Palpitation |
|  | Respiratory system disorders | - | - | Coughing, Dyspnoea, Nasopharyngitis, Upper respiratory tract infection |  |
|  | Urinary system disorders | Pyelonephritis acute | - | Urine abnormal, Urinary retention | Urinary retention |
|  | Reproductive disorders, female | - | - | Leukorrhoea, Menstrual irregularity | Leukorrhoea |
|  | Body as a whole - general disorders | Chest pain | - | Asthenia, Chest discomfort, Chest pain, Fatigue, Oedema generalized, Oedema peripheral, Pain, Pain in limb | Asthenia |
