# Supplementary material for: Comparison of safety profiles for dapagliflozin based on EMA and FDA safety issues: Challenges and future of post-marketing surveillance in Korea
Source: PLoS One. 2024 Nov 22;19(11):e0314363. doi: 10.1371/journal.pone.0314363 (PMC11584137; doi:10.1371/journal.pone.0314363)
Supplement: S2 File — AE, adverse event; IC, information component; KAERS, Korea adverse event reporting system; NOS, not otherwise specified; OHAs, oral hypoglycaemic agents; PRR, proportional reporting ratio; ROR, reporting odds ratio. (DOCX) [file pone.0314363.s003.docx]

**Supporting Information 2**

S1 Table: Empagliflozin KAERS signals meeting disproportionality analysis criteria

S2 Table: Ertugliflozin KAERS signals meeting disproportionality analysis criteria

**S1 Table: Empagliflozin KAERS signals meeting disproportionality analysis criteria**

| **AEs** | | **Number of AE reports** | | **Disproportionality analysis** | | | |
| --- | --- | --- | --- | --- | --- | --- | --- |
|  |  | **Empagliflozin** | **All other OHAs** | **PRR** | **ROR** | **IC** | $\boldsymbol{\chi}^{\boldsymbol{2}}$ |
| Skin and appendages disorders | | | | | | | |
|  | Pruritus genital | 72 | 596 | 7.10 | 7.39 | 2.34 | 339.06 |
| Musculo-skeletal system disorders | | | | | | | |
|  | Ligament disorder | 7 | 82 | 5.02 | 5.04 | 1.34 | 20.77 |
| Central & peripheral nervous system disorders | | | | | | | |
|  | Hypotonia | 8 | 81 | 5.81 | 5.83 | 1.57 | 28.99 |
| Vision disorders | | | | | | | |
|  | Conjunctivitis | 5 | 118 | 2.49 | 2.50 | 0.32 | 4.28 |
|  | Macula lutea degeneration | 3 | 30 | 5.88 | 5.89 | 1.29 | 11.04 |
|  | Vision abnormal | 10 | 239 | 2.46 | 2.47 | 0.50 | 8.33 |
| Psychiatric disorders | | | | | | | |
|  | Impotence | 4 | 81 | 2.90 | 2.91 | 0.47 | 4.76 |
| Gastro-intestinal system disorders | | | | | | | |
|  | Mouth dry | 22 | 544 | 2.38 | 2.40 | 0.66 | 16.97 |
|  | Tooth caries | 4 | 49 | 4.80 | 4.81 | 1.12 | 11.12 |
| Liver and biliary system disorders | | | | | | | |
|  | Cholelithiasis | 5 | 55 | 5.34 | 5.36 | 1.32 | 16.19 |
| Metabolic and nutritional disorders | | | | | | | |
|  | Diabetic ulcer | 3 | 55 | 3.21 | 3.21 | 0.53 | 4.32 |
|  | Ketosis | 12 | 45 | 15.67 | 15.78 | 2.85 | 130.22 |
|  | Lipid metabolism disorder NOS | 11 | 143 | 4.52 | 4.55 | 1.34 | 28.06 |
|  | Obesity | 3 | 8 | 22.04 | 22.08 | 2.65 | 43.83 |
|  | Thirst | 30 | 368 | 4.79 | 4.86 | 1.67 | 83.57 |
|  | Weight decrease | 83 | 1043 | 4.68 | 4.88 | 1.82 | 224.91 |
| Myo-, endo-, pericardial & valve disorders | | | | | | | |
|  | Angina pectoris | 13 | 252 | 3.03 | 3.05 | 0.86 | 16.88 |
|  | Cardiomyopathy | 3 | 38 | 4.64 | 4.65 | 1.00 | 7.94 |
| Urinary system disorders | | | | | | | |
|  | Cystitis | 14 | 229 | 3.59 | 3.62 | 1.11 | 24.76 |
|  | Micturition disorder | 3 | 45 | 3.92 | 3.92 | 0.78 | 6.12 |
|  | Micturition frequency | 41 | 540 | 4.46 | 4.55 | 1.64 | 103.00 |
|  | Nocturia | 8 | 185 | 2.54 | 2.55 | 0.48 | 7.19 |
|  | Polyuria | 31 | 393 | 4.64 | 4.71 | 1.64 | 82.31 |
|  | Urinary tract infection | 21 | 355 | 3.48 | 3.51 | 1.16 | 35.12 |
|  | Urine abnormal | 5 | 83 | 3.54 | 3.55 | 0.79 | 8.61 |
| Reproductive disorders, male | | | | | | | |
|  | Genital infection | 5 | 71 | 4.14 | 4.15 | 0.99 | 11.13 |
| Reproductive disorders, female | | | | | | | |
|  | Vaginitis | 33 | 266 | 7.29 | 7.42 | 2.23 | 159.88 |
| Neoplasms | | | | | | | |
|  | Uterine fibroid | 3 | 26 | 6.78 | 6.79 | 1.46 | 13.26 |
| Body as a whole - general disorders | | | | | | | |
|  | Malaise | 8 | 208 | 2.26 | 2.27 | 0.32 | 5.43 |
|  | Syncope | 7 | 91 | 4.52 | 4.54 | 1.21 | 17.84 |
| Resistance mechanism disorders | | | | | | | |
|  | Infection bacterial | 4 | 56 | 4.20 | 4.21 | 0.95 | 9.10 |
|  | Moniliasis genital | 3 | 32 | 5.51 | 5.52 | 1.21 | 10.13 |
|  | Sjogren's syndrome | 3 | 33 | 5.34 | 5.35 | 1.17 | 9.71 |
| Secondary terms - events | | | | | | | |
|  | Drug quality problem | 3 | 25 | 7.05 | 7.06 | 1.50 | 13.92 |
|  | Fall | 3 | 56 | 3.15 | 3.15 | 0.50 | 4.18 |
|  | Laceration | 9 | 100 | 5.29 | 5.31 | 1.49 | 28.76 |

**S2 Table: Ertugliflozin KAERS signals meeting disproportionality analysis criteria**

| **AEs** | | **Number of AE reports** | | **Disproportionality analysis** | | | |
| --- | --- | --- | --- | --- | --- | --- | --- |
|  |  | **Ertugliflozin** | **All other OHAs** | **PRR** | **ROR** | **IC** | $\boldsymbol{\chi}^{\boldsymbol{2}}$ |
| Metabolic and nutritional disorders | | | | | | | |
|  | Weight decrease | 4 | 1122 | 5.70 | 6.03 | 1.46 | 15.62 |
| Urinary system disorders | | | | | | | |
|  | Micturition frequency | 4 | 577 | 11.08 | 11.80 | 2.41 | 36.64 |
|  | Polyuria | 3 | 421 | 11.39 | 11.93 | 2.39 | 28.35 |
| Reproductive disorders, female | | | | | | | |
|  | Vaginitis | 3 | 296 | 16.20 | 17.00 | 2.89 | 42.48 |
